# Supplementary material for: Sulfated Hyaluronan Binds to Heparanase and Blocks Its Enzymatic and Cellular Actions in Carcinoma Cells
Source: Int J Mol Sci. 2022 May 2;23(9):5055. doi: 10.3390/ijms23095055 (PMC9102160; doi:10.3390/ijms23095055)

Table S1. Statistically corrected inter-fragment interaction energy (SCIFIE), inter-fragment interaction energy (IFIE) and its energy components electrostatic (ES), exchange repulsion (EX), charge transfer and higher order mixed term (CT+mix), and dispersion (DI). These were calculated as the interaction energy between each sugar residue of the tetrasaccharide and heparanase.

	**SCIFIE**	**IFIE**	**ES**	**EX**	**CT+mix**	**DI**
**hs-HA4**	**-2395.4**	-3123.0	-3122.1	212.8	-78.5	-135.2
(+1)	-633.5	-842.1	-830.7	80.7	-35.5	-56.6
(-1)	-562.6	-779.5	-774.8	38.1	-12.7	-30.1
(-2)	-635.3	-804.1	-817.9	58.6	-16.3	-28.4
(-3)	-521.4	-697.3	-698.8	35.5	-14.0	-20.1
**Dp4**	**-2500.5**	-3276.9	-3306.6	289.5	-99.3	-160.5
(+1)	-700.6	-920.0	-919.0	77.5	-30.7	-47.8
(-1)	-630.1	-809.6	-844.7	119.2	-29.0	-55.0
(-2)	-644.7	-809.3	-806.7	62.9	-26.4	-39.1
(-3)	-525.1	-738.1	-736.3	29.9	-13.2	-18.6

Table S2. Statistically corrected inter-fragment interaction energy (SCIFIE) between each sugar residue of the tetrasaccharide and two heparin binding domains (HBDs).

**HBD-1（Lys159-Asp171)**					
	Total	(+1)	(-1)	(-2)	(-3)
**hs-HA4**	-659.8	-65.7	-128.5	-339.1	-126.5
**Dp4**	-589.7	-101.0	-93.9	-214.9	-180.0
**difference**	-70.0	35.2	-34.6	-124.2	53.5

**HBD-2 (Gln270-Lys280)**					
	Total	(+1)	(-1)	(-2)	(-3)
**hs-HA4**	-553.4	-297.3	-112.9	-69.4	-73.8
**Dp4**	-526.0	-270.8	-116.8	-78.3	-60.0
**difference**	-27.4	-26.5	3.9	8.9	-13.8

Figure S1. Gel filtration of fluoresceine-labeled heparan sulfate degraded by human heparanase (huHpse). Inhibitors were added at different concentrations which are shown as μg/ml.

A: heparin (hep)

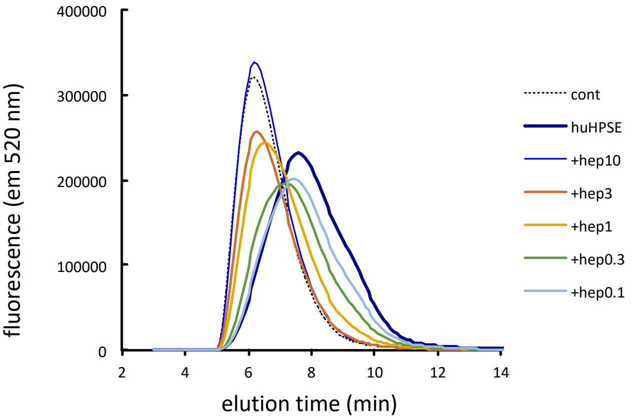

B: low-sulfated hyaluronan (ls-HA)

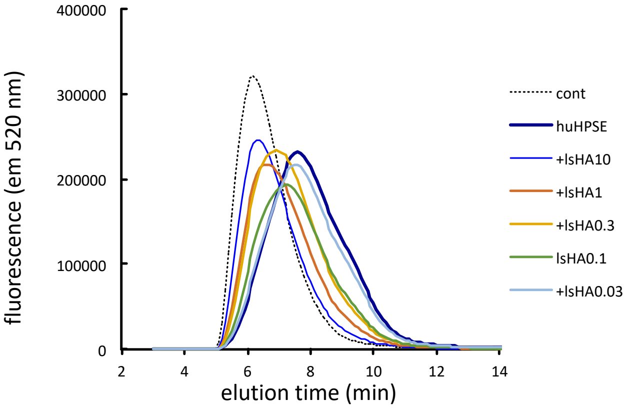

C: high-sulfated hyaluronan (hs-HA)

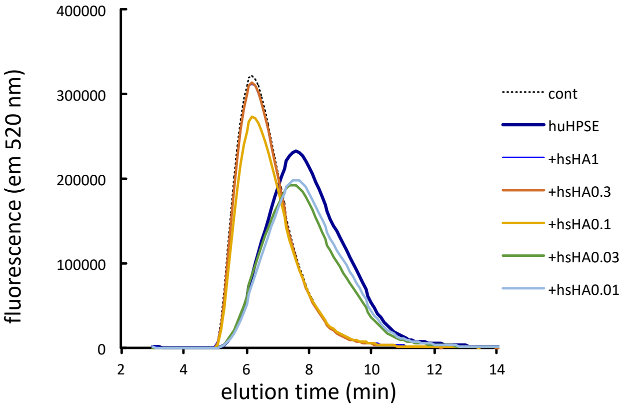

D: degradation ratio of panel A-C

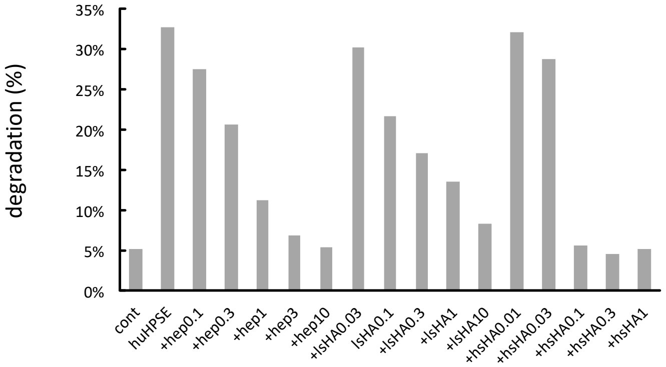

Figure S2. Gel filtration of fluorescently-labeled HS degraded by muHpse. Inhibitors were added at different concentrations which are shown as μg/ml.

A: heparin

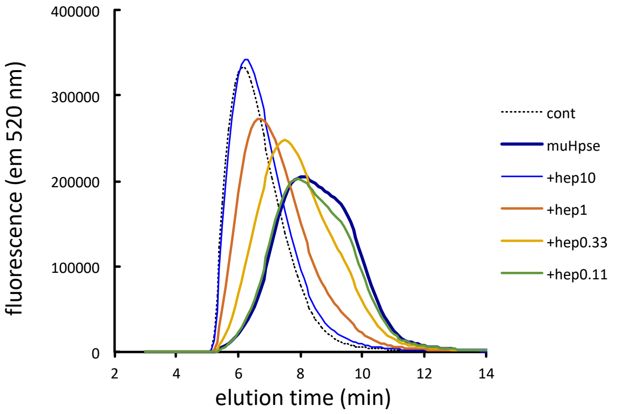

B: ls-HA

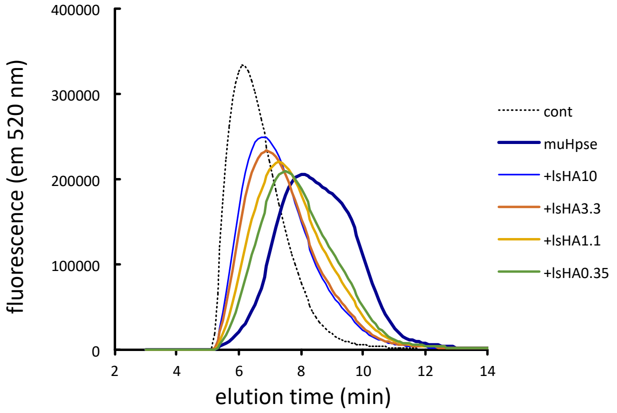

C: hs-HA

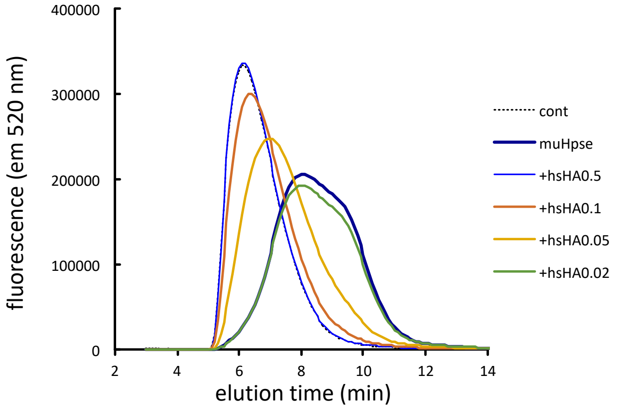

D: degradation ratio of panel A-C

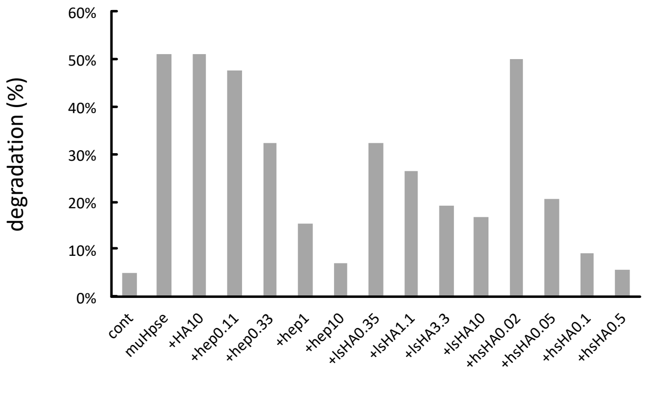

Figure S3. Structures of hs-HA4 and Dp4 tetrasaccharides. The delimiter lines indicate the fragmentation positions.

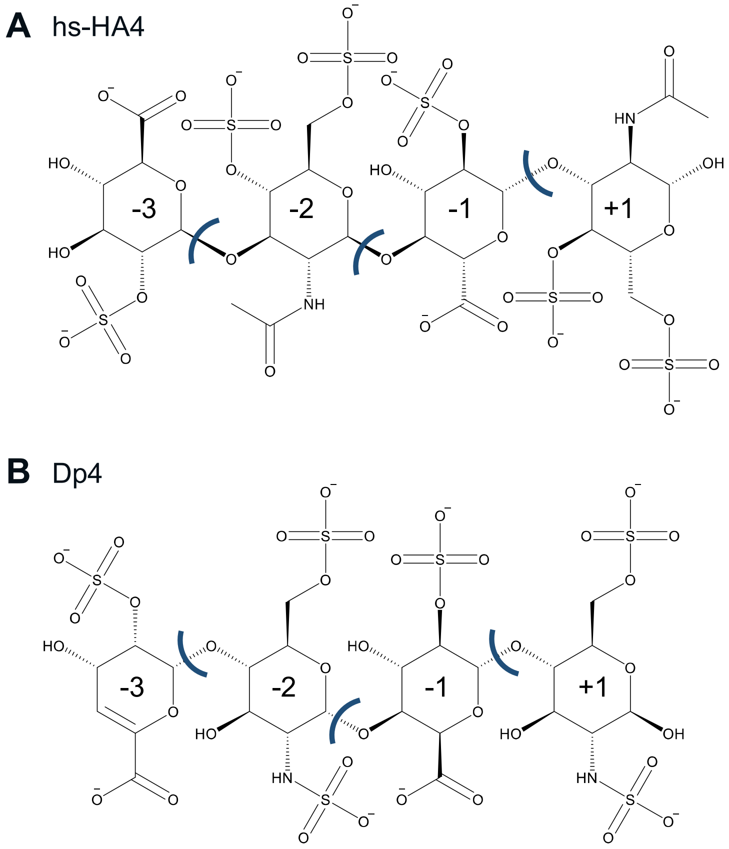

Figure S4. Location of tetrasaccharides and heparin binding domains (HBDs). Atoms in the tetrasaccharides are indicated by sticks, and HBD-1 and HBD-2 are indicated by pink and green ribbons, respectively.

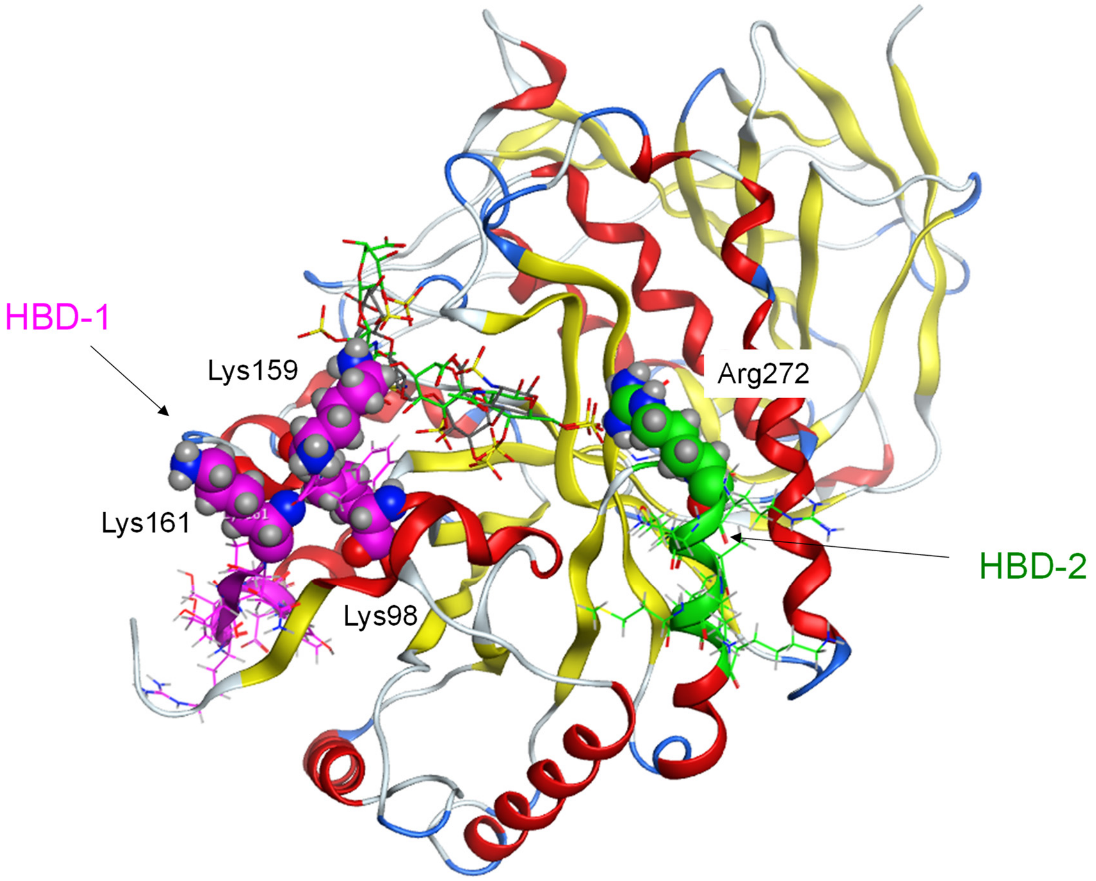

Figure S5. Interaction of the 6-*O*-sulfate group of the (-2) sugar residue of the tetrasaccharides with Lys98 of huHpse.

A: hs-HA4

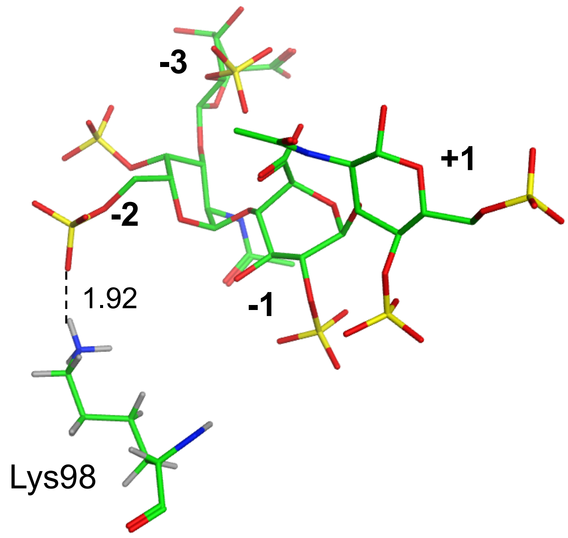

C: Superimposed image

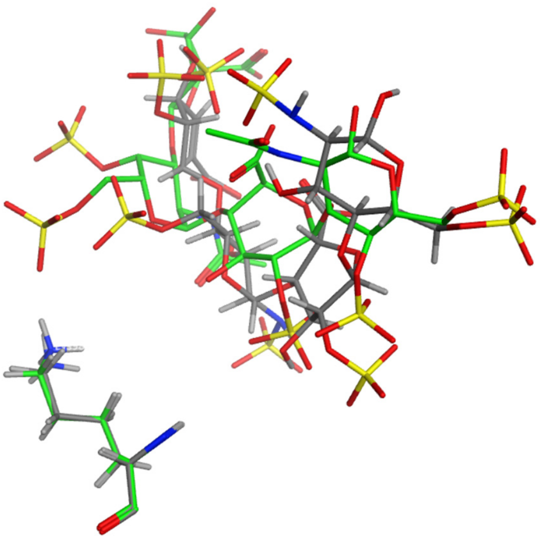

B: Dp4

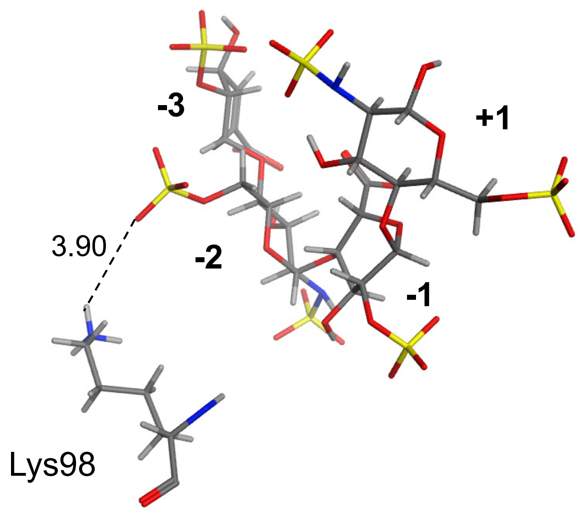

Supplement: Supplementary file 1 [file ijms-23-05055-s001.zip › ijms-1674093-supplementary.pdf]
